# Mutational Profiling of Non-Small-Cell Lung Cancer Resistant to Osimertinib Using Next-Generation Sequencing in Chinese Patients

**DOI:** 10.1155/2018/9010353

**Published:** 2018-03-11

**Authors:** Keke Nie, Haiping Jiang, Chunling Zhang, Chuanxin Geng, Xiajuan Xu, Ling Zhang, Hao Zhang, Zhongfa Zhang, Ketao Lan, Youxin Ji

**Affiliations:** ^1^Department of Radiotherapy, Qingdao Cancer Hospital, Qingdao 266042, China; ^2^Department of Oncology, The Affiliated Hospital of Qingdao University, Qingdao 266002, China; ^3^Department of Oncology, The Affiliated Qingdao Central Hospital of Qingdao University, Qingdao 266042, China; ^4^Oncology Genemic, Geneplus Beijing, Beijing 100010, China

## Abstract

**Purpose:**

To identify the somatic mutated genes for optimal targets of non-small-cell lung cancer after resistance to osimertinib treatment.

**Patients and Methods:**

Study patients all had advanced lung adenocarcinoma and acquired resistance to osimertinib as a second- or third-line treatment. These patients had harboring EGFR T790M mutation before osimertinib treatment, which was confirmed by Amplification Refractory Mutation System (ARMS) PCR or Next-Generation Sequencing (NGS). After resistance to osimertinib treatment, tumor tissue was collected by core needle biopsy. DNA was extracted from 15 × 5 um sliced section of formalin-fixed paraffin-embedded (FFPE) material and NGS was done. The genetic changes were analyzed.

**Results:**

A total of 9 Chinese patients were studied, 5 females and 4 males, age 51–89 years. After progression with osimertinib treatment, core needle biopsy was performed and next-generation sequencing was performed. Nine patients had harboring 62 point mutations, 2 altered gene copies, 2 amplifications, and 1 EML4-ALK gene fusion. No MET or HER2 amplification was found in this cohort study. Nine patients still maintained initial EGFR 19 del or L858R activating mutations, while 7 of them kept EGFR T790M mutations. Among the 7 patients, 5 had secondary EGFR C797S and/or C797G mutations, which all happened in the same allele with T790M mutation. All patients were treated with targets therapies, chemotherapy, or best supportive care (BSC) in accordance with NGS genetic results and patients' performance status; 7 of them are still alive and 2 of them died of disease progression at last follow-up.

**Conclusions:**

EGFR C797S/G mutation and the same one presented on the same allele with EGFR T790M mutation were the most common mutation feature and played a key role in resistance to osimertinib in Chinese patients with NSCLC. Tumor cells losing T790M mutation and maintaining EGFR activating mutation might benefit from first-generation EGFR-TKI treatment.

## 1. Introduction

Epidermal growth factor receptor (EGFR) T790M mutation is the most common genetic change for patients of non-small-cell lung cancer (NSCLC) harboring EGFR after resistance to first-generation EGFR tyrosine kinase inhibitor (TKI) [1]. The substitution of threonine with methionine at amino acid position 790 (T790M), which reduces the ability of ATP-competitive reversible EGFR-TKI binding to EGFR tyrosine kinase domain, results in cancer cells resistant to gefitinib and erlotinib [1]. Osimertinib (Tagrisso, AZD9291, AstraZeneca) is the only FDA approved drug for lung cancer patients harboring EGFR T790M mutation. After a median of 9.6–11.0 months' remission with osimertinib treatment, tumors will inevitably have progress. Although a lot of studies had been done, the molecular mechanisms of resistance are not yet fully understood [2, 3].

Next-generation sequencing (NGS) is a cost-effective technology capable of screening several genes simultaneously [4]. It is commonly used nowadays for sequencing mutated tumor genes with tumor tissue or plasma to identify and classify molecular subtypes, to address the unmet need for new drug targets in its category [5]. The mechanism of resistance to osimertinib or other third-generation EGFR-TKI was extremely complicated, and the reported results of mutation sites and/or mutation rates were much different among studies. Phenotype transformation, EGFR new point mutation, pathways activation, or targets loss were the strongest possibilities. Most studies reported that C797S mutation happened in 20–30% of patients after osimertinib initiation [6, 7]. The EGFR C797S mutation conferred resistance to third-generation EGFR-TKI. C797S mutation had been identified in cis or in trans with T790M mutation in tumor specimens from patients who experienced treatment failure with third-generation EGFR-TKIs. C797S and T790M mutation in trans were sensitive to first-generation plus third-generation EGFR-TKI but in cis they would be resistant to all [8]. Therefore, to elicit the mutated driver genes after resistance to third-generation EGFR-TKI is critically important.

## 2. Material and Methods

Patients enrolled in the study all had histologically confirmed metastatic lung adenocarcinoma. EGFR T790M mutation was confirmed by tumor tissue or serum, which was tested by the ARMS PCR or Next-Generation Sequencing (NGS) before osimertinib treatment. Patients were all treated with osimertinib with a dose of 80 mg oral daily after resistance to gefitinib or erlotinib treatment. Osimertinib acquired resistance was confirmed by CT or PET-CT scan according to RECIST 1.1 [9]. Core needle biopsy (CNB) guided by CT scan was performed. DNA was extracted from 15 × 5 um sliced sections of FFPE tumor tissue. Tumor area was evaluated and confirmed by pathologist. In order to ensure adequacy of sequences and mutation detection, at least 20% tumor area on each slice was set as a minimum. 10 ml blood was drawn and centrifuged for sequencing control and for germline genes mutation test. NGS was performed with HiSeq3000/HiSeq4000 Illumina techniques. 4278 exons of 288 common genes; intron, promoter, and fusion of 38 genes; and coding area of 728 genes were tested for somatic mutations. 11 germline mutations were also tested. The ultra-deep coverage of genes of interest was 1,000x for tumor tissue and 10,000x for serum.

This study was approved by the Ethics Committee of Affiliated Qingdao Central Hospital of Qingdao University, and Informed consent to reveal patients' medical history for publishing was obtained before submitting this manuscript.

## 3. Results

**Figure 1:**
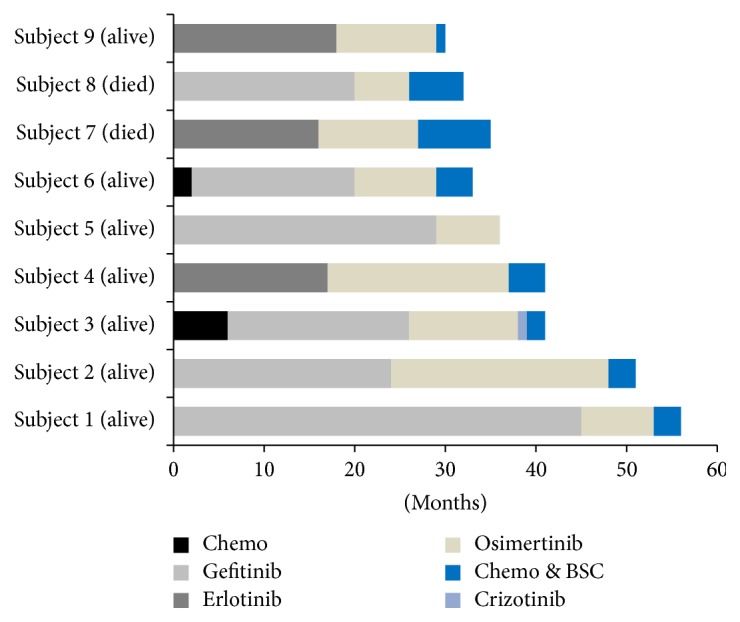
Treatment and outcomes.

A total of 9 Chinese patients were studied. There were 5 female patients and 4 male patients, median age 66-year, range 51–89. All patients were core needle biopsied and adenocarcinomas were confirmed by pathologists; there was no SCLC transformation found. Tissues obtained sources for testing and genetic testing methodologies were listed (Table 1). There were 62 point mutations, 2 altered gene copies, 2 amplifications, and 1 EML4-ALK gene fusion found in these 9 patients (Table 2). No MET or HER2 amplification was found in this cohort study. All the 9 patients still maintained initial EGFR 19 del or L858R activating mutations; meanwhile, 7 of them kept EGFR T790M mutations. Furthermore, among the 7 patients, 5 had secondary EGFR C797S and/or C797G mutations, which all happened in the same allele with T790M mutation. Two patients had EGFR C797G 2389T>G mutations; 1 had EGFR C797S 2390G>C mutation; 1 had EGFR C797S 2389 T>A and 2390 G>C 2 points mutations; and 1 had EGFR C797S 2389 T>A, 2390 G>C 2 points and C797G 2389 T>G mutations. Subjects #1 and #5 had acquired EGFR T790M mutations tested by NGS, but lost them after osimertinib treatment. There were 9 point mutations before osimertinib treatment, which increased to 22 point mutations after resistant to osimertinib. Most pre-osimertinib point mutations could be tested after osimertinib treatment with the exception of T790M mutation and WSCD2 mutation (Table 3). The above 2 patients were treated back with gefitinib, one having had stable disease for 3 months, the other one just started. Subject #3 had a low mutation rate of EML4-ALK fusion and simultaneously had EGFR L858R, T790M mutation, and C797G mutation and was treated with crizotinib but had progression in 1 month. He was treated with pemetrexed for 2 cycles and had a confirmed partial response. Subject #6 had harboring high MAF of EGFR EXON21 (c.2500G>T, p.V834L), 19 del, TP53, T790M, and C797G mutations (91.1%, 85.5%, 85.2%, 61.5%, 36.6%, resp.) and was resistant to gefitinib combined with osimertinib but had stable disease with cisplatin plus pemetrexed chemotherapy. Subject #7 had EGFR C797S (2389 T>A, 2390 G>C) mutations and was treated with erlotinib, osimertinib, and cetuximab but had progression in 2 months and died 6 months later. Subject #8 had EGFR 19 del and T790M and C797S/G mutations and was treated with BSC and died after 6 months. The other 3 patients were in chemotherapy or BSC at last follow-up on December 15, 2017 (Figure 1).

## 4. Discussion

Activation of epidermal growth factor receptor (EGFR) triggers antiapoptotic signaling, proliferation, angiogenesis, invasion, and metastasis, which lead to development and progression of NSCLC [10]. Inhibition of EGFR by tyrosine kinase inhibitors such as gefitinib and erlotinib has increased tumor response and prolonged patients' survival. However, acquired resistance will finally happen, with a progression-free survival (PFS) of around 9–13 months [11, 12]. The substitution of threonine with methionine at amino acid position 790 (T790M), as the second mutation in EGFR, is the most common resistance mechanism and is detected in tumor cells from more than 50–60% of patients after disease progression. This mutation enhances ATP affinity and reduces the ability of ATP-competitive reversible EGFR-TKI binding to EGFR tyrosine kinase domain, which results in cancer cells resistant to gefitinib and erlotinib [1]. Osimertinib is an oral, potent, irreversible EGFR-TKI and inhibits kinase activity of EGFR sensitive mutation and T790M resistant mutation [13, 14]. Osimertinib for second-line treatment in EGFR T790M positive NSCLC was associated with a response rate of 61% and PFS of 9.6 months [13]. But the mechanism of osimertinib resistance is much complicated [6, 7]. Third-generation EGFR-TKIs (WZ 4002, rociletinib, osimertinib) have pyrimidine structure and covalent bond with EGFR Cys797 at ATP binding pocket. Cys797 point mutation (C797S/G/R) intercepts 3rd-generation EGFR-TKIs covalent binding to Cys797 at ATP binding pocket, which results in drug resistance [15, 16]. Thress et al. [17] reported results of 15 patients NGS after osimertinib progression: 6/15 cases acquired the C797S mutations, 5/15 cases maintained the T790M mutations but did not acquire the C797S mutation, and 4/15 cases lost the T790M mutations. In our results, all 5 acquired C797S/G mutations, patients still maintained EGFR T790M mutations, and C797S/G and T790M mutations were in the same allele. In in vitro study, tumor cells harboring C797S mutation without T790M mutation are still sensitive to quinazoline-based EGFR inhibitors like gefitinib or erlotinib, and, harboring EGFR C797S in trans with T790M mutations are sensitive to a combination of first- and third-generation EGFR-TKI and in cis are resistant to all current EGFR inhibitors [18, 19].

HER2 or MET amplifications were also reported in osimertinib-resistant NSCLC patients. EGFR T790M mutation and HER2 amplification appeared to be mutually exclusive while MET amplification occurred with or without EGFR T790M mutation [20, 21]. It was hypothesized that tumor heterogeneity, HER2, or MET subclones initiated an independent pathway for tumor progression [17, 22].

There were 2 patients who had acquired low MAF PIK3CA mutations; 1 patient had acquired NOTCH3, IGF1R, and PIK3B mutations. These point mutations are all associated with osimertinib use and might correlate with drug resistance [23], which needs further study to elicit new methods to overcome it.

Small-cell lung cancer transformation is rare in NSCLC patients resistant to EGFR-TKIs treatment. It might preexist before first-/second-/third-generation EGFR-TKIs treatment [24]. So, rebiopsy is critically important in the diagnosis of SCLC transformation after resistance to 3rd-generation EGFR-TKIs.

In our study, 2 patients had been tested by NGS before and after osimertinib treatment. There were 5 point mutations excluding EGFR activating mutation and EGFR T790M mutation before osimertinib treatment. After resistance to osimertinib, 16 new point mutations were found except loss of T790M mutation and WSCD2 mutation. This phenomenon indicated that the more the lines of EGFR-TKIs therapies were initiated, the more the point mutations would happen. These 2 patients were treated back with gefitinib; 1 had stable disease for 3 months. It was indicated that 1st-generation EGFR-TKI could be used in patients who lost T790M mutation but maintained EGFR 19 del or L858R activating mutation. 1 patient had low rate EML4-ALK gene fusion (0.9%), EGFR 19 del (2.8%), and T790M (4.6%) and C797G (2.6%) mutations and was treated with crizotinib and progressed in 1 month but responded to chemotherapy thereafter. It might be that tumor cells with EML4-ALK gene fusion were too low. Subjects #6 and #7 had high MAF of EGFR 19 del, T790M, and C797S or C797G mutations and were resistant to 1st- and 3rd-generation combination. So, chemotherapy is the optimal treatment for C797S/G in cis with T790M mutation before new drugs can overcome it.

In this study, 5/9 (55.6%) patients had acquired C797S/G mutations, all maintained with T790M mutation in the same allele. In these 5 patients, 3 times of c.2389T>G, 2 times of c.2389T>A, and 3 times of c.2390G>C point mutations were found. The C797S/G mutations were maintained with T790M mutation in cis, and C797S/G mutations happened to be higher than previous report [17]. In the 9 patients, there were a total of 62 point mutations, 2 altered copy numbers, and 2 amplifications, and 1 patient with 2 points C797S (2389 T>A, 2390 G>C) mutations and 1 patient had 2 points C797S and C797G mutations, which made the mechanism of resistance to osimertinib more complicated.

## 5. Conclusions

Genetic mutation is much complicated after osimertinib treatment in EGFR positive non-small-cell lung cancer. EGFR C797S/G mutation and the same one presented on the same allele with EGFR T790M mutation were the most common mutation feature and played a key role in resistance to osimertinib in Chinese patients with NSCLC. Tumor cells losing T790M mutation and maintaining EGFR activating mutation might be benefit from first-generation EGFR-TKI treatment. To elicit a chemical to overcome EGFR C797S/G point mutation with T790M mutation in the same allele is critically important.

## Figures and Tables

**Table 1:** Patients' characteristics.

#	Sex	Age (yrs)	EGFR type	Tissues and genetic testing			
				Before osimertinib		After osimertinib	
1	F	62	L858R	Lung	NGS	Pleura	NGS
2	M	85	19 del	SL	ARMs-PCR	Lung	NGS
3	M	66	19 del	Bone	ARMs-PCR	Lung	NGS
4	F	79	L858R	Lung	ARMs-PCR	Lung	NGS
5	F	89	19 del	Lung	NGS	Lung	NGS
6	F	66	19 del	Serum	ARMs-PCR	Bone	NGS
7	M	56	19 del	Lung	ARMs-PCR	Lung	NGS
8	F	75	19 del	Pleura	ARMs-PCR	Pleura	NGS
9	M	51	19 del	Lung	ARMs-PCR	Lung	NGS
Median		66					

SL denotes supraclavicular lymph node.

**Table 2:** Mutation types and mutant allele fraction (MAF) (%).

#1		#2		#3		#4		#5		#6		#7		#8		#9	
Mutation	Rate	Mutation	Rates	Mutation	Rates	Mutation	Rates	Mutation	Rates	Mutation	Rates	Mutation	Rates	Mutation	Rates	Mutation	Rates
L858R	63.2	19 del	48.6	19 del	2.8	L858R	22.2	19 del	2.1	19 del	85.5	19 del	51.9	19 del	24.3	19 del	65.68
IL7R	31.5	T790M	15.1	T790M	4.6	T790M	2.2	PIK3CA	1.8	T790M	61.5	T790M	32.5	T790M	18.7	T790M	46.04
TENM3	27.1	JAK1	13.7	C797G	2.6	NOTCH3	27.5	TP53	1.7	C797G	36.6	C797S T>A	18.4	C797S T>A	6	C797S G>C	11.4
CCND3	14.3	NF2	11.4	EML4-ALK	0.9	IGF1R	15.1	RNASEL	1.2	EGFR 834>L	91.1	C797S G>C	0.8	C797S G>C	5	TP53	35.62
ROS1 (6737 G>A)	2.9	TET2	10.5	PIK3CA	0.9	ZNF512B	10.5	TET2	0.8	TP53	85.2	EGFR2134T>A	0.3	C797G	2.2	C11orf30	17.1
FGFR3	1.9	FCGR2A	18.1			TMPRSS2	10.5	MAP3K1	0.8			TP53	36.7	CTNNB1	38.8	FLT4	12.01
XPC	1.5					MLL3	7.8					RB1	20.5	MED12	34.8		
ATM	1.5					PIK3CB	6.7					PIK3CA	0.7	APC	21.9		
FAM135B	1.2					EGFR L406Q	6.6					BRCA1	0.6	SCF1R	4.6		
PALB2	1.1					FLT4	4.9					ALK	0.6	FCGR2R	2.5		
MLL	1					ERBB3	3.3					JAK1	0.5				
ROS1 (1611A>G)	1					FAT1	3.2					ROS1 2495T>A	0.4				
CDKN2A	0.2					HPS3	3.2										
Altered copy						GNAS	2.5										
CDKN2B DEL	0.3					CLB	2.2										
CDKN2A DEL	0.2					CDKN1A	1.9										
						SMO	1.9										
						ROBO3	1.7										
						PTCH1	1.7										
						CDK12	1.4										
						MDM2	1.2										
						FOXL2	1.1										
						Amplification											
						MDM2	3.8										
						CDK4	5.0										

**Table 3:** Mutation types and mutant allele fraction (MAF) (%) changes.

#1				#5			
Before osimertinib		After osimertinib		Before osimertinib		After osimertinib	
Mutation	Rate	Mutation	Rates	Mutation	Rates	Mutation	Rates
L858R	42.9	L858R	63.2	19 del	9.6	19 del	2.1
IL7R	19.1	IL7R	31.5	T790M	1.1	PIK3CA	1.8
T790M	5.7	TENM3	27.1	TP53	7.11	TP53	1.7
CCND3	5.8	CCND3	14.3			RNASEL	1.2
WSCD2	5.1	ROS1 (6737 G>A)	2.9			TET2	0.8
FGR3A	4.1	FGFR3	1.9			MAP3K1	0.8
		XPC	1.5				
		ATM	1.5				
		FAM135B	1.2				
		PALB2	1.1				
		MLL	1				
		ROS1 (1611A>G)	1				
		CDKN2A	0.2				
		Altered copy					
		CDKN2B DEL	0.3				
		CDKN2A DEL	0.2				
